# Detecting diseases of neglected seminal vesicles using imaging modalities: A review of current literature

**Published:** 2016-05

**Authors:** Gautam Dagur, Kelly Warren, Yiji Suh, Navjot Singh, Sardar A. Khan

**Affiliations:** 1 *Department of Physiology and Biophysics, SUNY at Stony Brook, New York, USA.*; 2 *Department of Urology, SUNY at Stony Brook, New York, USA.*

**Keywords:** *Seminal vesicles*, *Infertility*, *Hematospermia*, *Transrectal ultrasound*

## Abstract

Seminal vesicles (SVs) are sex accessory organs and part of male genitourinary system. They play a critical role in male fertility. Diseases of the SVs, usually results in infertility. Diseases of the SVs are extremely rare and are infrequently reported in the literature. We address the current literature of SV pathologies, symptoms, diagnosis, and treatment options. We review the clinical importance of SVs from PubMed. The current imaging modalities and instrumentation that help diagnose SV diseases are reviewed. Common pathologies including, infection, cysts, tumors, and congenital diseases of the SVs are addressed. Many times symptoms of hematospermia, pain, irritative and obstructive lower urinary tract symptoms, and infertility are presented in patients with SV diseases.

## Introduction

Seminal vesicles (SVs) are part of male genitourinary system. Male genital organs include the penis, testes, excretory genital ducts, vas deferens, SVs, prostate, and bulbourethral glands. SV is considered accessory gland which plays a major role in male fertility. Male genital organs work collectively to produce and excrete semen, composed of mature spermatozoa (1). SV pathophysiology and imaging modalities have not been well described in major textbooks. SVs diseases are extremely rare and are infrequently reported in literature, however the importance of SV diseases is emphasized in this article. Here we address the current literature of SV pathologies, symptoms, diagnosis, and treatment options. 

The purpose of this study is to bring awareness of critical importance of SV diseases to the clinicians attention.

**Clinical examination of seminal vesicles**

Two parts of clinical examination include symptoms (Table I) and physical diagnosis.

**Table I:** Clinical symptoms secondary to seminal vesicles diseases (Irritative and obstructive lower urinary tract symptoms

Odynorgasmia (2)	Pelvic Pain	Groin Pain (8)
Painful ejaculation	Flank Pain	Anal Tenesmus (9)
Hematospermia	Abdominal Pain	Intra-abdominal Swelling (10)
Decreased Ejaculate	Constipation (4)	Proctalgia Fugax (11)
Hematuria	Epididymitis	Diarrhea (12)
Oligospermia	Emphysematous-Epididymitis (5)	Pneumaturia (12)
Azoospermia	Syncope (6)	Inguinal Hernia (13)
Seminal Hyperviscosity (3)	Rectal Obstruction (7)	

**Physical examination of seminal vesicles**

Digital rectal examination (DRE) can potentially help obtain clinical index of suspicion when diseases of SVs are present. However DRE is not the best means to suspect SVs disease. SVs are infrequently palpable when the bladder is distended. Palpation is also dependent on the length of index finger as they are located retrovesical and above the prostate gland. DRE is a good indicator of enlarged SV cysts (14).

**Imaging modalities**

**Transrectal ultrasound**

Transrectal ultrasound (TRUS) should be the first-line modality for genitourinary tract imaging because it is minimally an invasive imaging modality, inexpensive, high availability, decreased need for sedation, dynamic evaluation capabilities, extension of physical diagnosis and no radiation is involved. Normal transverse imaging of SVs shows elongated mass found superior to the prostate. Oblique imaging, shows SVs joining with the terminal portion of vas deferens, forming the ejaculatory duct (15). TRUS is an extension of DRE when clinical symptoms are suggestive of seminal vesicle diseases.

**Computed tomography**

Computed tomography (CT) produces a three-dimensional image of internal body structure, constructed using by a series of plane cross-sectional images. Contrast-enhanced CT, shows SVs as fluid-filled structures, with a thin septa. This modality remains the most helpful in recognizing many SV abnormalities (15).

**Magnetic resonance imaging**

Magnetic resonance imaging (MRI) is another helpful form of modality to recognize SV abnormalities. Using low signal intensity T1-weighted and high signal intensity T2-weighted images shows normal SVs as elongated fluid-filled structure with thin septa (15).

**Positron emission tomography**

Positron emission tomography (PET) may localize tumors of the SVs (16).

**Diagnostic instrumentation**

Two current diagnostic instrumentations used for SVs include, transurethral seminal vesiculoscopy and cystoscopy (17, 18).

**Embryology**

During the 5^th^ wk of gestation, the ureteric bud develops from the mesonephric duct. During the 7^th^ wk of gestation, the testes develop and differentiate the male genital system. SVs, initially starts as a bulbous swelling of distal mesonephric duct during the 12^th^ wk gestation. SVs are retro-vesicle to the uro-genital sinus (19).

**Anatomy**

SVs are bilateral glands. They are 5-7 cm long. SVs are rounded at the superior position and tapered inferiorly. SVs are found dorsal to the bladder, and inferior and lateral to the vas deferens. Bilateral arrangement of the SVs, results in a “V” shape. Ureters are located superior and in between SVs. SVs are located superior to prostate gland. SVs lie at the inferior-most aspect of recto-vesical space in pelvic cavity. SV ducts marge with ampulla of vas deferens and form the ejaculatory duct which opens into the prostatic urethra (1).

**Physiology**

SVs contains many highly granular cells, which produce a yellowish, alkaline fluid. This fluid contains fructose, proteins, and vitamin C. Testosterone level plays a significant role on these cells. They dictate the size and activity levels of the cells. The fructose of fluid provides energy for the spermatozoa motility, . This fluid accounts for 50% of ejaculate total volume. Rest the seminal fluid volume comes from the prostate gland, ampulla of vas deferens and lesser amounts from the bulbourethral glands, Cowper’s glands (20).

**Diseases of the Seminal Vesicles**

**Congenital**

**Seminal vesicle agenesis**

SV agenesis is a congenital anomaly, where there is a complete or partial absence of one or both SVs. This anomaly may result in infertility (21). Patients are generally asymptomatic. Only symptom patients demonstrate is infertility (21, 22). First line modality to diagnosis of patients with SV agenesis is TRUS (22). CT is used to confirm the TRUS findings (23). No treatment options are available for the SV agenesis correction.

**Zinner syndrome**

Zinner syndrome is presentation of SV cysts with ipsilateral renal agenesis and ectopic ureter insertion into SV cyst. This tends to asymptomatic, and is diagnosed when the patients report infertility later in their life. Haddock *et al* reported a rare case, where the patient presents with pelvic pain during ejaculation. DRE was not able to identify the issue, but suggested dilated epididymis and vas deferens. CT and MRI revealed the presence of a cyst near the right SV and absent right kidney. Transrectal cyst aspiration was used to remove the cyst. Injection of a sclerosing agent revealed an ectopic ureter (24). Robotic or robotic-assisted laparoscopic resection was used to remove the ectopic ureter (24, 25). Congenital SV cyst is usually categorized with Zinner syndrome (26).

**Seminal vesicle hypoplasia**

Hypoplasia of the SVs can be unilateral or bilateral. Raviv *et al* used TRUS for patients who presented with azoospermia, determining bilateral hypoplasia of SVs (27). However, MRI provided precise diagnosis of SV defect, better than TRUS (28).

**Seminal vesicles secondary to cystic fibrosis**

In patients with cystic fibrosis, SVs may be absent, hypoplastic, and/or with lack of prostaglandin and fructose (29).

**Cystic**

**Seminal megavesicles**

Seminal megavesicles, or giant cysts of SVs, present secondary to autosomal dominant polycystic kidney disease (ADPKD). Reig *et al* identified patients with ADPKD have an average diameter of SV tubule 4.2 mm, ranging 1.7-30 mm, whereas patients without ADPKD and cysts have a diameter of SV tubule 3.1 mm, ranging from 1.7-6.8 mm (30). Patients with seminal megavesicles may present with infertility. TRUS was enough to diagnose the patient presenting with azoospermia secondary to seminal megavesicles (31).

**Seminal vesicle hydatid cyst**

SVs Hydatid cyst is a rare disease. It may go undetected due to nonspecific symptoms. Vasileios *et al* presented a case of patient with urinary retention secondary to hydatid cyst, or echinococcal cyst, of the SVs. Diagnosis was done using TRUS, CT, and MRI. Surgical excision of cyst is the best option but must be done with caution to avoid puncturing and parasite spillage in retroperitoneal space (32). Other symptoms seen secondary to hydatid cyst includes dysuria, nocturia, frequency, and tenesmus, as seen in a case presented by Tuygun *et al* (33).

**Seminal vesicle hemorrhage**

Hematospermia is a main symptom secondary to SVs hemorrhage. Hasegawa *et al* conducted a study to determine the etiology of hematospermia. MRI was useful to find any abnormalities. Hasegawa *et al* concluded one reason for hematospermia could be hemorrhage of the SVs (34).

**Hypotonic seminal vesicles**

Hypotonic SVs can result in infertility. Infertility is due to diabetic autonomic neuropathy of SVs, decreasing the secretion of seminal fluids. La Vignera *et al* determined that duration of diabetes correlates to neuropathy level. It was concluded that patients with diabetes greater than 15 yrs, had a greater atony of SVs due to neuropathy (35). Potentially treating diabetes early on, can prevent long term infertility. 

**Infection**

**Seminal vesicle abscess**

SVs abscess is a rare pathology that is rarely encountered (36). It is an infection that develops on SVs due to bacterial or viral microorganisms. Patients suffering from SVs abscess present with many uro-genital symptoms (37). Abscesses of the SVs may develop secondary to a surgical procedure due to infection. SVs abscess may be developed secondary to vasectomy, tuberculosis, and prostate biopsy (38-41). There are different diagnostic modalities present to diagnosis SVs abscess, CT, MRI, but TRUS should be primary means of diagnosis (41-44). Cui *et al* described another modality, transurethral seminal vesiculoscopy, which is used to diagnosis and treat hematospermia secondary to SVs (17). Drainage of abscess is the most common means of treatment (44).

**Seminal vesiculitis**

Seminal vesiculitis is the SVs inflammation. It is a common disease of male urogenital tract. Its pathogenesis is unclear, but the lack of semenogelin I secretion is believed to be the cause of seminal vesiculitits, as it has antibacterial properties to prevent bacterial inflammation (45). Patients with seminal vesiculitis present with hematospermia, discomfort and pain in lumbosacral or perineal region, irritative and obstructive urinary symptoms, decreased semen volume, and/or azoospermia (46). CT and MRI diagnose the complication and transurethral surgery corrected the issue (46). TRUS can diagnosis cases of seminal vesiculitis, as well (47). Furuya *et al* determined that patients with urethritis are likely to have seminal vesiculitis, suggesting a close relationship between them (48). It is also known that epididymitis is possible along with seminal vesiculitis (49).

**Seminal vesicle cyst infection**

SV cyst infection occurs because of bacterial infection and can result in many complications. Palmer *et al* reported a case of patient presenting symptoms of perineal pain and fever. The patient had come in earlier with methicillin-sensitive staphylococcus aureus bacterium, and was treated with antibiotics. CT revealed an expansion of a SV cyst, and MRI was used to confirm the diagnosis as infected cyst. Cyst was drained and it was determined that methicillin-sensitive bacterium was the cause of infection and the patient was discharged on vancomycin (50). Xu *et al* study revealed hematospermia due to SV cyst infection. Transvesical removal of mass was an effective surgical procedure to alleviate the disease (51).

**Solid**

Most benign and malignant tumors of SVs appear to be solid on TRUS. However, the cystic component may be present (Table II). 

**Table II:** Benign and malignant tumors of seminal vesicles

**Benign**	
Mixed Epithelial-Stromal Tumor (52)	Cystic Schwannoma (69)
Primitive Neuroectodermal Tumor (54)	Mammary-Type Myofibroblastoma (71)
Leiomyoma (56)	Schwannoma (73)
Phyllodes Tumor (58)	Primary Myxoid Solitary Fibrous Tumor (75)
Basal Cell Hyperplasia (53)	Neurilemmoma (77)
Cystadenoma (61)	Hemangiopericytoma (79)
Malacoplakia (63)	Fibromuscular Hyperplasia (81)
Stromal Tumor (65)	Benign Mesenchymoma (83)
Multiocular Adenomyoma (67)	
**Malignant**	
Intraepithelial Neoplasia (53)	Primary Leiomyosarcoma (70)
Primary Seminal Vesicle Carcinoma (55)	Primary Diffuse Large B-Cell Lymphoma (72)
Malignant Solitary Fibrous Tumor (57)	Primary Burkitt Lymphoma (74)
Primary Squamous Cell Carcinoma (59)	Primary Rhabdomyosarcoma (76)
Primary Yolk Sac Tumor (60)	Transitional Cell Carcinoma In Situ (78)
Primary Bilateral Carcinoma (62)	Primary Seminoma (80)
Primary Extragastrointestinal Stromal Tumor (64)	Primary Angiosarcoma (82)
Primary Adenocarcinoma (66)	Primary Carcinoid Tumor (84)
Neuroendocrine Carcinoma (68)	Round-Cell Sarcoma (85)

**Primary adenocarcinoma of seminal vesicle**

Adenocarcinoma is a malignant tumor formed from glandular structures in epithelial tissue. Adenocarcinoma of SVs (ASVs) is considered an extremely rare malignancy. Secondary spread of this disease is common. There are very few cases reported worldwide, approximately fewer than 100 cases (86). Etiology of this pathology is unclear, but patients present symptoms of obstructive uropathy, hematuria, and hematospermia (87-89). Diagnosis of ASVs is difficult, as they are negative for prostate-specific antigen and prostate-specific acid phosphatase. Immunophenotype of ASVs are positive for cancer antigen 125 and 7 (55). Primary diagnostic steps include, DRE identifies as a mass, which requires further examination, and TRUS and biopsy (89, 90). MRI, CT with contrast, after histopathology, determine the mass as SVs adenocarcinoma . Best course of treatment for ASVs is postoperative chemotherapy and hormonal therapy (55, 91, 92). Prognosis of ASVs is very poor, and metastasis usually results in death (55, 92).

**Adenosarcoma-like tumor of seminal vesicle**

Adenosarcoma-like tumor of the SVs is a rare pathology. Patients with this pathology present with increased frequency and painful defecation, acute urinary retention due to bladder outlet obstruction, and hematospermia (93, 94). Chheda *et al* described a recurrent case of adenosarcoma-like SVs tumor , the patient complained of increased frequency of micturition and dysuria (93). CT is the best means of diagnosis for this pathology (93, 94). Exploratory laparotomy with wide excision of the mass, and chemotherapy are used to treat the patients (93).

**Seminal vesicles amyloidosis**

SVs amyloidosis is the build-up of amyloid proteins in SVs commonly found in older men, and is more prominent with age (95). It is associated with hematuria, hematospermia, and prostatitis (95-98). Amyloidosis of SVs is commonly found after TRUS guided prostate biopsy (96). Yang *et al* presented seven patients with SV amyloidosis. Patients that underwent immunohistological study, were positive for amyloid P, Therefore, it was not systemic amyloidosis (96, 99). Though it is possible to develop systemic amyloidosis in SVs (100). TRUS should be the first line of diagnostic modality, as it can show SV amyloidosis (101). T2-weighted MRI is another imaging modality that can assist in diagnosis of this pathology (97). To treat amyloidosis, laparoscopic resection can eliminate the pathology (101).

**Seminal vesicle angiosarcoma**

Angiosarcoma of the SVs is an extremely rare and malignant tumor. This cancer arises from inner lining of blood vessels. Chang *et al* described a patient presenting with groin pain and pain in left lower quadrant. CT used to reveal the mass in SV. TRUS-guided biopsy confirms angiosarcoma. Angiosarcoma treatment of SV is involved neoadjuvant chemotherapy, which decreases the size of mass, and surgical resection of tumor (102).

**Calculi of seminal vesicle**

Calculi of the SVs are an extremely rare pathology. A stone develops in SVs which results in obstruction. A common symptom seen in patients with SVs calculi is hematospermia (17). Painful ejaculation can be seen following SV calculi (103). To determine the cause of hematospermia, TRUS was used because of its noninvasive nature (104). Other imaging modalities used to diagnose the cause of hematospermia are endorectal MRI and CT, though it is not common in studies reported (105, 106). It is possible for a calculi to develop in SV after transurethral resection of ejaculatory duct, as described by Vellayappan *et al* possibly secondary to urinary reflux (106). A method to treat, as well as diagnosis calculi of SVs is transurethral seminal vesiculoscopy, when combined with finasteride it is a safe method to treat hematospermia secondary to SV calculi, as described by Cui *et al* (17). Laparoscopy is another means to treat SV calculi (107).

**Calcification of seminal vesicles**

Calcification of SVs was first described in 1906 (108). This is not a common pathology and its incidence is unknown. It is usually seen secondary to radiation, diabetes mellitus, tuberculosis, schistosomiasis,and more. Calcification can occur unilaterally or bilaterally (109-113). Calcification of SVs can present as hematuria, dysuria, hematospermia, and flank pains (109, 111). Another symptom presented in patients is azoospermia (114). TRUS revealed a lesion with SVs calcification . It was confirmed using CT (115). There is no specific treatment of SVs calcification . Treatment should be designed for underlying cause of calcification (110).

**Cystadenoma of seminal vesicles**

SVs cystadenoma is a benign tumor, an extremely rare pathology. Most tumors of SVs tend to be malignant. Cystadenoma are generally asymptomatic (116). Arora *et al* presented a case of a patient with lower abdominal pain and obstructive urinary symptoms. DRE revealed a soft, painless mass but used TRUS and MRI to diagnosis the pathology. Surgical excision was conducted to remove the mass (117). Lee *et al* reported another case of cystadenoma of SVs, using CT to diagnose the mass (116).

**Seminal vesicle leiomyoma**

Leiomyoma is a benign tumor of smooth muscle found on SVs. This is a rare pathology. Miyalima *et al *reported a case of patient presenting with lower abdominal discomfort (56). Abdominal discomfort and pain of the lower back are common symptoms of SVs leiomyoma , and urinary symptoms (56, 118, 119). Diagnosing leiomyoma of SVs is involved CT and/or MRI of abdomen in almost all cases (56, 118, 120-122). In the cases found, TRUS was not used to diagnose the pathology. Surgical approach is the only way to treat the appearance of this pathology, and conduct follow-up to determine if the patient is disease free (56, 122).

**Schwannoma of seminal vesicles**

Schwannoma of SVs is a tumor that develops on Schwann cells of SV. It is a rare disorder. Patients with Schwannoma of the SVs can be asymptomatic, or present with hydronephrosis, lower urinary tract symptoms, or lower abdominal pain (69, 123-125). In many cases, diagnosing can be accidental, as in case reported by Fievet *et al* (126). Mass can be detected using TRUS, CT, and MRI (69, 123, 124). Arun *et al* diagnosed the mass using cystoscopy after TRUS and CT did not provide visible separation of prostate and SV (123). Surgical excision, laparoscopic surgery, of the tumor is the first line approach for treatment (69, 123).

**Neuroendocrine carcinoma of seminal vesicle**

Neuroendocrine carcinoma of SVs (NCSVs) are primary tumor of SVs that are malignant. Prognosis of NCSV is extremely poor and patients usually die by a disease (68). A unique manifestation of NCSV is Lambert Eaton syndrome (LES). LES is an autoimmune disease that leads to degeneration of neuromuscular junction (127). Kreiner *et al* reported a case of patient with SV mass, detected using CT and PET for LES evaluation. TRUS-guided biopsy identified a poorly differentiated neuroendocrine carcinoma. Treatment suggested for pathology was chemotherapy and surveillance (16). Other symptoms seen secondary to NCSV is obstructive uropathy (128).

**Seminal vesicle phyllodes tumor**

Phyllodes tumor, also known as mixed epithelial-stromal tumors, encompasses low, intermediate and high-grade tumors. this tumor is benign in most cases, sometimes it may become malignant (52). Some symptoms presented secondary to phyllodes tumor of SVs include flank pain, on either side depending on which SV has the mass, urinary obstruction, and hematospermia (58, 129, 130). TRUS, CT, and MRI can aid in identifying a large mass on seminal vesicle. Seminal vesiculectomy remove the tumor from SVs (129).

**Seminal vesicle primitive neuroectodermal tumor**

SV primitive neuroectodermal tumor is rare, with an unclear origin. DRE and TRUS can detect a mass present but further diagnostic imaging is necessary. De Paula *et al* reported a case of patient showing a solid mass after CT. After declining TRUS-biopsy, the patient returned complaining of rectal stricture and urinary obstructive symptoms. Biopsy suggested primitive neuroectodermal tumor. Patient was treated with two cycles of chemotherapy, and then underwent laparotomy to excise the mass (131).

**Secondary carcinoma of seminal vesicles**

Diseases arising from different organs, may extend to SVs, from distant or local regions of the body. Disease that metastatize to SVs include, melanoma, renal cell carcinoma, testicular tumor, hepatocellular carcinoma, prostate carcinoma, rectal carcinoma, and bladder carcinoma (132-138).

**Seminal vesicles fistula**

SVs fistula is secondary to rectal adenocarcinoma and may present with rare symptoms of diarrhea and pneumaturia. Treatment of this rare complication includes administration of metronidazole (12). Patients with SVs fistula present themselves secondary to iatrogenic resection of cancer, Crohn’s disease, and neoplastic infiltration (12, 139).

## Conclusion

SVs are part of male genitourinary systems, and play a critical role in aiding the motility of sperm, therefore are necessary for male fertility. Diseases of SVs can result in male infertility. Patients suffering from diseases of SVs present with a diverse number of symptoms like hematospermia, pain, irritative and obstructive lower urinary tract symptoms. There are a variety of categories that incorporate the SVs diseases . These categories are congenital, cystic, infection, solids, and fistula. We addressed the methods of diagnosis of SVs diseases, both imaging modalities and instrumentational.

## Conflict of interest

The authors declare they have no conflict of interest.
